# Fourier Transform Infrared with Attenuated Total Reflectance Applied to the Discrimination of Freshwater Planktonic Coccoid Green Microalgae

**DOI:** 10.1371/journal.pone.0114458

**Published:** 2014-12-26

**Authors:** Guilherme Pavan de Moraes, Armando Augusto Henriques Vieira

**Affiliations:** Laboratory of Phycology, Department of Botany, Universidade Federal de São Carlos (UFSCar), São Carlos, São Paulo, Brazil; University of Quebect at Trois-Rivieres, Canada

## Abstract

Despite the recent advances on fine taxonomic discrimination in microorganisms, namely using molecular biology tools, some groups remain particularly problematic. Fine taxonomy of green algae, a widely distributed group in freshwater ecosystems, remains a challenge, especially for coccoid forms. In this paper, we propose the use of the Fourier Transform Infrared (FTIR) spectroscopy as part of a polyphasic approach to identify and classify coccoid green microalgae (mainly order Sphaeropleales), using triplicated axenic cultures. The attenuated total reflectance (ATR) technique was tested to reproducibility of IR spectra of the biological material, a primary requirement to achieve good discrimination of microalgal strains. Spectral window selection was also tested, in conjunction with the first derivative treatment of spectra, to determine which regions of the spectrum provided better separation and clustering of strains. The non-metric multidimensional scaling (NMDS), analysis of similarities (ANOSIM) and hierarchical clusters (HCA), demonstrated a correct discrimination and classification of closely related strains of chlorophycean coccoid microalgae, with respect to currently accepted classifications. FTIR-ATR was highly reproducible, and provided an excellent discrimination at the strain level. The best separation was achieved by analyzing the spectral windows of 1500–1200 cm^−1^ and 900–675 cm^−1^, which differs from those used in previously studies for the discrimination of broad algal groups, and excluding spectral regions related to storage compounds, which were found to give poor discrimination. Furthermore, hierarchical cluster analyses have positioned the strains tested into clades correctly, reproducing their taxonomic orders and families. This study demonstrates that FTIR-ATR has great potential to complement classical approaches for fine taxonomy of coccoid green microalgae, though a careful spectrum region selection is needed.

## Introduction

Recent developments in the use of marker genes provided great advances and profound changes in systematics, especially for microorganisms [1]. However, classification remains unsolved in some groups because of the lack of reliable morphological traits, or the absence of enough genomes sequenced or good marker genes. This is the case of coccoid green algae, a widespread group in inland waters. Identify and classify coccoid green algae is an extremely complex and laborious task. Associated with major problems regarding the definition and separation of species in this group, there is a huge number of taxa and few studies focused in this problematic group, rendering it hard to classify and position species within and across hierarchical clusters [2].

Different approaches have been proposed to discriminate species and resolve a natural phylogeny for the group: the *morphological species*, whose definition is distorted by phenotypic plasticity and convergent evolution [3]–[5], and *phylogenetic species*, which uses marker genes, such as 18S ribosomal RNA (18S rDNA), internal transcribed spacer (ITS) and plastid rubisco large subunit (rbcL), among others. While the phylogenetic approach is considered most reliable, it leads to some divergences in identification and delimitation of species, owing to markers being either too conservative or too variable [4]–[6].

The taxonomy of coccoid green algae turned out to be so complex that it has been suggested that only a polyphasic approach could be fruitful [2]. Features such as ecophysiological and biochemical characteristics could help to find natural taxonomic groups for these microalgae. In this context, the Fourier transformed infrared (FTIR) spectroscopy analysis might be extremely useful. The FTIR is a technique that applies interferometric modulation of infrared (IR) radiation to capture the chemical composition of the integral sample in a way that the whole chemical composition is monitored at the same time throughout the analysis [7]. The resulting spectrum is an interaction/superposition of spectra from each individual chemical component, rendering a unique species-specific pattern. FTIR spectra of biological samples, such as microalgal cells, can be used to discriminate them, as these spectra can be understood as complete phenotypic and genotypic fingerprints of the sample [7].

This technique not only weights the differences in a few genes or diacritic morphological characteristics, but assesses the whole genome of the microorganisms and chemical composition of cells components, with an emphasis on what is really being expressed in that particular moment. Moreover, the FT-IR technique is extremely fast, simple to perform, inexpensive and also does not require any solvent or reagent other than water and the sample, resulting in a non-destructive technique.

The first studies applying IR technics in the study of microorganisms date from the 1950s [8], [9], but at that time, the early equipment's performance was lower, and the computational capacity limited the data analysis [7]. Thus, this technique would be used again for this objective only in 1970's [10]. The first systematic study of microorganisms by FT-IR was conducted by Helm, Naumann and collaborators [11]–[14], who demonstrated that the absorption spectra in the mid-infrared region could be used to identify and discriminate bacteria. Few studies were carried out on the microalgae with identification purposes [15], [16], many more focusing on monitoring biomass composition changes in response to stress [16]–[22].

These previous works on microalgae systematics tested the suitability of different spectrum regions combinations for chemometric separation of several strains of marine microalgae and cyanobacteria, and achieved good separations between large taxonomical groups (divisions, classes, orders and families) using spectra acquired through transmittance technique from samples deposited on suitable windows [15], [16].

One problem with this technique is related to the film thickness of the film sample. Variations in film thickness, common in the window deposition technique, impair spectral quality and reproducibility from spectra acquired through transmittance technique. The procedure normally used to cope with this problem is to divide the whole spectrum by the intensity of the amide I band after determining the minimum biomass required to produce a homogeneous film with a good signal-to-noise ratio, without band saturation [16]. In other words, this approach is essentially normalizing the spectrum with respect to biomass in the film, assessed by the protein present in the sample (inferred by the amide I band), in order to compensate for thickness variation.

Here, we propose the use of Attenuated Total Reflectance (ATR) to circumvent this difficulty. With this technique, only attenuated radiation interacts with the sample with always the same penetration depth, eliminating band saturation and problems with film thickness variation, effectively removing the main source of spectrum variations, at the same time that enables an easy and fast sample preparation procedure.

Therefore, this study aimed to collect highly reproducible FTIR - ATR spectra, eliminating the variations obtained with the transmittance technique, in order to determine whether it is possible to discriminate chemometrically not only large taxonomical groups, but closely related coccoid green algae species and contribute, in a polyphasic framework, to resolve the identification and phylogeny of this problematic group.

## Materials and Methods

### Strains and culturing

Organisms of Chlorophyceae (Chlorophyta), order Sphaeropleales, family Selenastraceae (*sensu* Krienitz & Bock [2]) were the main focus. The strains used were obtained from the Inland Water Microalgae Culture Collection at Federal University of São Carlos (CCMA-UFSCar in Portuguese acronymic, in São Carlos - Brazil), listed here with identification numbers after their respective names. The strains were classified with morphological characters, according to the current classification (as can be seen in Algae Base website, http://www.algaebase.org, and in reference [2]): *Ankistrodesmus densus* Korshikov, 1953 (003), *Ankistrodesmus densus* Korshikov, 1953 (128), *Ankistrodesmus densus* Korshikov, 1953 (239), *Ankistrodesmus fusiformis* Corda ex Korshikov, 1953 (333), *Selenastrum bibraianum* Reinsch, 1866 (047), *Selenastrum bibraianum* Reinsch, 1866 (241), *Selenastrum gracile* Reinsch, 1866 (350) and *Monoraphidium komarkovae* Nygaard, 1979 (353) (Selenastraceae, Sphaeropleales, Chlorophyceae, Chlorophyta); *Desmodesmus communis* (E. Hegewald) E. Hegewald, 2000 (030), *Coelastrum* cf *sphaericum* Nägeli, 1849 (060) and *Scenedesmus ecornis* (C.G. Ehrenberg ex J. Ralfs, 1845) R.H. Chodat, 1926 (088) (Scenedesmaceae, Sphaeropleales, Chlorophyceae, Chlorophyta); *Chlamydomonas clorastera* Ettl 1968 (009) (Chlamydomonadaceae, Chlamydomonadales, Chlorophyceae, Chlorophyta) and *Micrasterias pinnatifida* Ralfs, 1848 (089) (Desmidiaceae, Desmidiales, Zygnemophyceae, Charophyta).

The strains , all axenic, were cultured in triplicate in 1.8 L of WC medium [23] in 2 L Boeco flasks, aerated with compressed air, filtered by a 0.22 µm filter, flowing at 0.05 L.min^−1^ per liter of culture, under light of intensity 300 µmol photons m^−2^. s^−1^, at controlled temperature of 25±1°C. For *Micrasterias pinnatifida* (089), 10^−6^ M iron-EDTA solution was added to the WC medium [24].

Growth curves were prepared by monitoring optical density at wavelength 682 nm (except for strains 089, 128 and 241, with readings at wavelength 680 nm), and the *in vivo* chlorophyll *a* content, measured by Trilogy fluorometer from Turner Designs, in order to follow the culture growth and perform the FTIR-ATR measurements in the late-exponential growth phase, as recommended by Kansiz et al [15]. The reason for this recommendation is that the difference between spectra of the same culture due to changes in intracellular content as the culture ages, collected few days apart from one another, is minimized in this phase. Mean optical density and chlorophyll *a* content of triplicates were analyzed to decide when to harvest the cultures.

Cells were harvested and washed in WC medium lacking nitrogen, phosphorus, vitamins and micronutrients was performed by double centrifugation (3500×g for 7–10 minutes on 50 mL falcon tube). Cultures were thus concentrated, from 1.8 liters to approx. 15 mL, frozen, lyophilized and stored at −20°C until FTIR analyses were performed.

### Acquisition of spectra and sample preparation

Spectra were collected on a Shimadzu IRAffinity-1 FTIR spectrophotometer with an air cooling light ceramic light source and DLATGS pyroelectric detector, controlled by a PC running IRsolution software that accompanies the equipment. Absorbance spectra were collected in the range 4000–630 cm^−1^, with 4.0 cm^−1^ resolution and 128 co-added and averaged spectra, with apodization by triangular function.

Samples were prepared from the lyophilized biomass of the cultures in the form of a homogeneous film deposited on a HATR (Horizontal Attenuated Total Reflectance, Pike Technologies) trough plate accessory, which has an embedded ZnSe crystal and a beam incidence angle of 45°, providing space for ten reflections of the beam over the sample coating the crystal.

The lyophilized biomass was suspended in deionized water at 3 mg mL^−1^, and 0.5 mL was pipetted onto the ATR plate, which was gently shaken to distribute the suspension evenly over the entire crystal. These preparations were dried for approximately 18 min under a heated fan at mild temperature (approx. 45°C) and wind speed. Quadruplicate absorption spectra (analytical replicates) were collected from different film coatings for each of the 3 culture replicate.

### Chemometrics analysis

Before the chemometrics analysis, it was necessary to pre-treat the spectra. The pre-treatment, previously determined by iterative method, was intended to reduce random variations, such as those caused by differentiated dispersion between samples and absolute variations due, for instance, to differences in the biomass deposited on the plate [25], with the aim of improving the discriminating power of the technique.

Such data enhancement can be achieved by taking the first derivative of the spectrum [10], [26]. This has the benefit of highlighting spectral features that were not readily recognized in the original spectra [27]. This treatment was performed with IRsolution software, selecting a 9 – point first-order differentiation (number of points also determined iteratively).

Next, a region of the spectra was selected that was later subdivided into five windows, matching band assignments to biomolecules, to perform and assess the analysis. The region selected was based on earlier works that aimed for microalgae discrimination [7], [15], [16], and ranged from 1800–630 cm^−1^. The windows were defined as: window I 1800–1700 cm^−1^, window II 1700–1500 cm^−1^, window III 1500–1200 cm^−1^, window IV 1200–900 cm^−1^ and window V 900–630 cm^−1^. The whole region and various combinations of the windows were tested. Analysis was carried out with R software [28] (packages ‘MASS’ and ‘vegan’).

The separation between strains was estimated at first by comparing stress values in Kruskal's NMDS analysis (non-metric multidimensional scaling), calculated from the Euclidean distance matrix, and R values in the ANOSIM analysis (analysis of similarities) of species, calculated from the same matrix. To improve the discrimination of strains and to generate a possible classification dendrogram for them, HCA (Hierarchical Cluster Analysis) was carried out using single linkage algorithm and Euclidean distance.

## Results

**Figure 1:**
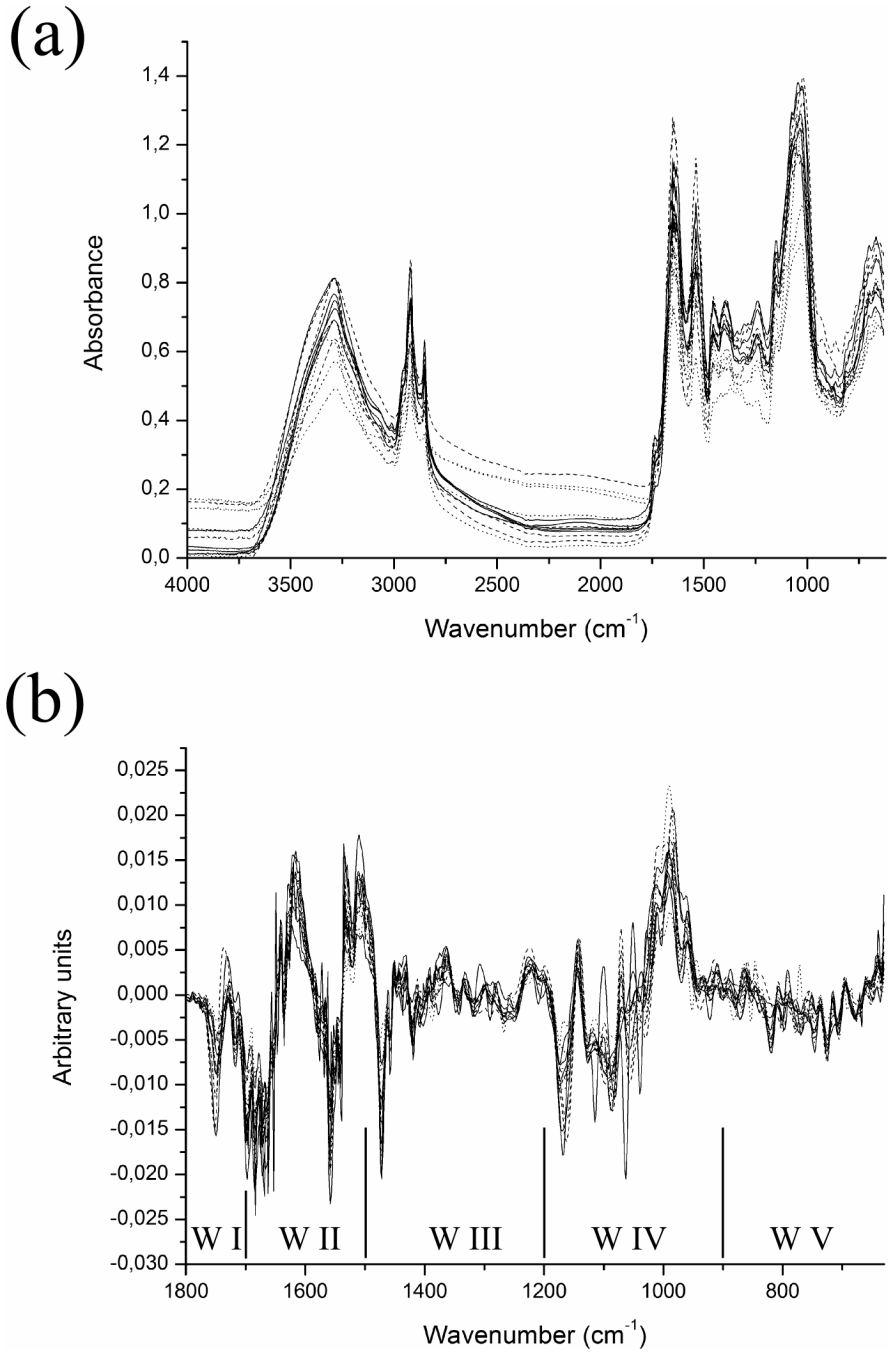
Representative raw and treated spectra. (a) Raw total spectra of all strains tested, from 4000–630 cm^−1^ and (b) enlargement of spectral region 1800–630 cm^−1^ of all strains transformed by a 9-point first derivative, with the 5 spectral windows indicated. W I: 1800–1700 cm^−1^; W II: 1700–1500 cm^−1^; W III: 1500–1200 cm^−1^; W IV: 1200–900 cm^−1^ and W V: 900–675 cm^−1^.

Raw spectra from all strains used in this study are shown superposed in Fig. 1a. An enlargement of the region used in chemometrics, with the 9-point first derivative performed, stating the spectral windows which subdivide it, is shown in Fig. 1b. The biomolecular assignments of these windows, are as follows [7], [14]: (I) window 1800–1700 cm^−1^, assigned to C = O bonds of esters and carboxyl groups of DNA/RNA; (II) window 1700–1500 cm^−1^ relates to amide groups I, II, III in proteins; (III) window from 1500–1200 cm^−1^ is a mixed region, with superposed information about C = O bonds in organic acids, phosphodiesters and amide III in proteins; (IV) window 1200–900 cm^−1^, assigned to C-O-C and C-O bonds in polysaccharides and PO_2_^-^ asymmetric and symmetric stretching vibrations; and (V) window 900–675 cm^−1^, regarded as the “true fingerprint region”, unspecific to any compound or functional group. For a more detailed table of compound assignments to spectrum regions, refer to the cited works.

It should be stressed that we did not use whole true fingerprint region (900–600 cm^−1^), because of the absorbance range of the crystal in the ATR accessory. Specifications of the manufacturer state that the minimum wavenumber for this accessory is 630 cm^−1^. However, we noted variation rising around 650 cm^−1^ towards lower wavenumbers in the raw spectra, so we applied chemometrics analysis of data starting from 675 cm^−1^, although spectral acquisition went down to 630 cm^−1^.

**Figure 2:**
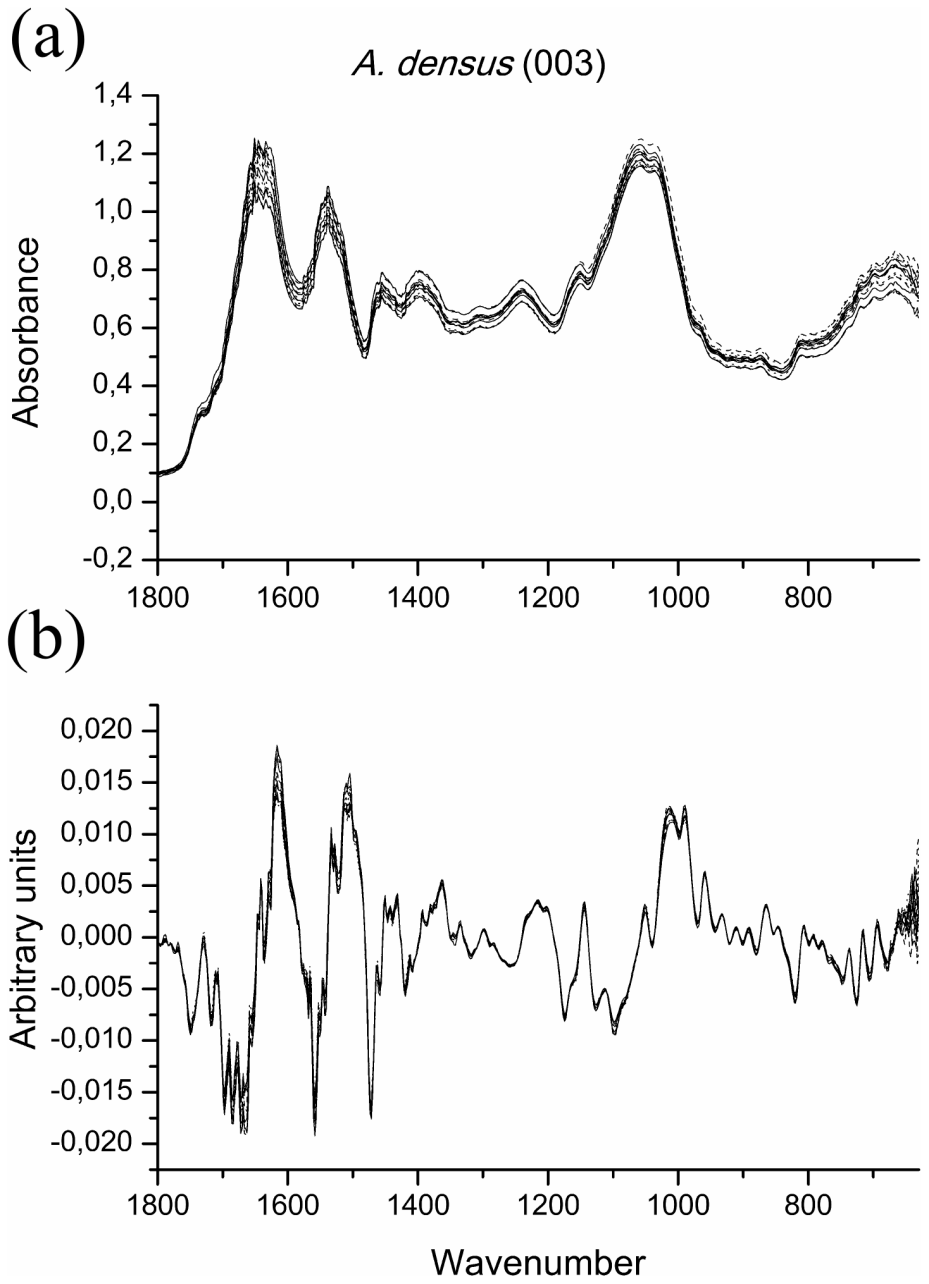
Comparison of (a) raw spectra and (b) their 9-point first derivative treatment, for *Ankistrodesmus densus* (003). Region shown is 1800–630 cm^−1^, representing the results for all other strains but *C. clorastera* (009), *D. communis* (030) and *C.* cf. *sphaericum* (060).

**Figure 3:**
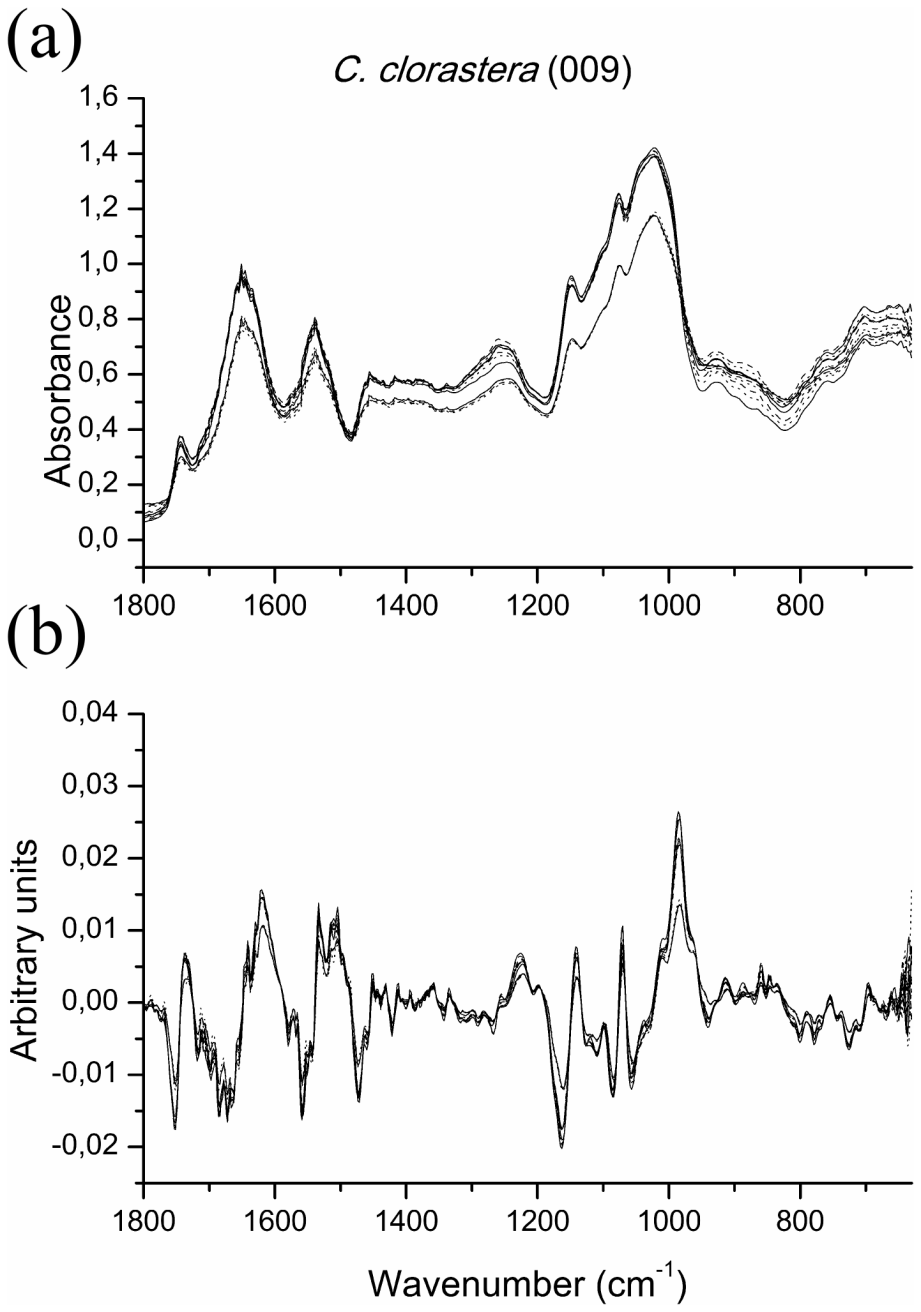
Comparison of (a) raw spectra and (b) their first derivative treatment, for *Chlamydomonas clorastera* (009). Region shown is 1800–630 cm^−1^ for both, showing the differences from Fig. 2 and representing the similar results achieved for the strains *Desmodesmus communis* (030) and *Coelastrum* cf. *sphaericum* (060).

It can be seen that the raw spectrum has a very good reproducibility, even without any data treatment (Fig. 2). However, strains *C. clorastera* (009), *D. communis* (030) and *C.* cf. *sphaericum* (060) showed a higher degree of variation on their raw spectra between the replicate cultures over the entire region presented, although the main features are still visibly discernible. These results are shown for strain *C. clorastera* (009), representing the similar results obtained for strains *D. communis* (030) and *C.* cf. *sphaericum* (060) (Fig. 3) (see Supporting Information, S1 and S2 Figs., for the raw and treated spectra figures for all other strains).

These variations also occur, to a lesser extent, for all other strains. This is the reason for carrying out pre-treatment of the data, which is used to remove the variations between replicates, due to differentiated dispersion of samples and differences in biomass [15], [25], [26]. The pre-treatment consisted in using 9-point first derivatives of the spectra, thus improving the reproducibility of the spectra and enhancing their spectral differences (Figs. 2 and 3).

By comparing stress values from the NMDS analysis (Table 1), we found that the best combination of spectral windows (lowest stress value) for strain separation in a two-dimensional representation consisted of windows III and V (10.391). In a three-dimensional representation, the lowest stress value came from the combination of spectral windows IV and V (4.528).

**Table 1:** Non-metric multidimensional scaling (NMDS) stress values for models with two and three dimensions and analysis of similarities (ANOSIM) R-values and their significance (p) for strains differentiation of five spectral windows combinations.

	NMDS stress values		ANOSIM	
Spectral windows	2 dimensions	3 dimensions	R	p
1800–675	10,543	6,306892	0,9359	<0,001
1700–675	10,418	5,582564	0,93	<0,001
1500–675	10,862	4,923337	0,9184	<0,001
1200–675	10,783	4,527858	0,9067	<0,001
1800–1700_1500_675	11,395	6,258952	0,9308	<0,001
1800–1200_900–675	12,174	6,795809	0,9387	<0,001
1700–1200_900–675	11,192	6,520498	0,9349	<0,001
1500–1200_900–675	10,391	6,354459	0,9542	<0,001

**Figure 4:**
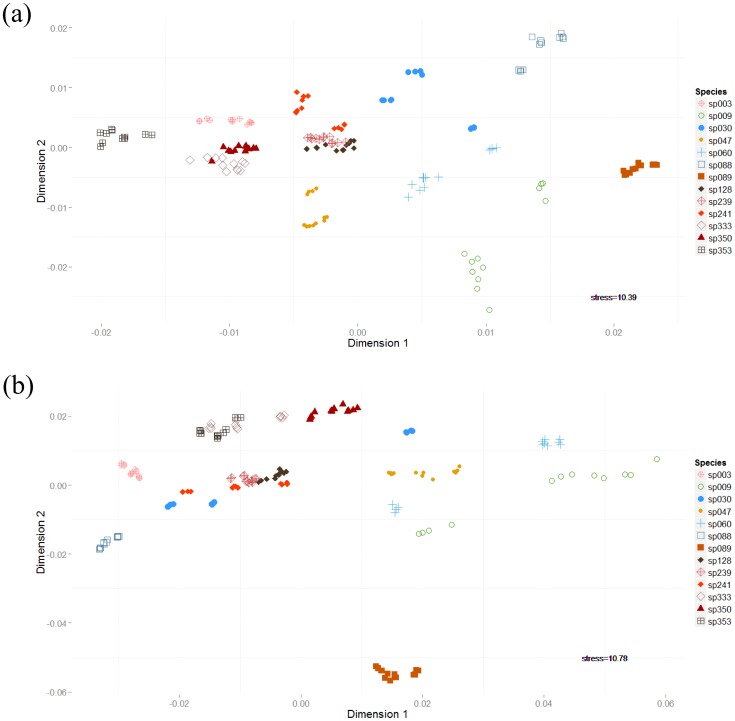
Assessment of strain separation by NMDS of the 9-point first derivative spectra. Two different spectral window combinations are shown: (a) window III and V and (b) window IV and V. Strains of microalgae: *Ankistrodesmus densus* (003), *Chlamydomonas clorastera* (009), *Desmodesmus communis* (030), *Selenastrum bibraianum* (047), *Coelastrum* cf *sphaericum* (060), *Scenedesmus ecornis* (088), *Micrasterias pinnatifida* (089), *Ankistrodesmus densus* (128), *Ankistrodesmus densus* (239), *Selenastrum bibraianum* (241), *Ankistrodesmus fusiformis* (333), *Selenastrum gracile* (350) and *Monoraphidium komarkovae* (353).

The ANOSIM analysis (also Table 1) indicated that spectral windows III and V (R = 0.9542, p<0.001) provided a better discrimination than windows IV and V (R = 0.9067, p<0.001). In fact, data from spectral windows IV and V resulted in the lowest ANOSIM R-value of the five tested combinations of windows, the remaining combinations showing intermediate values (Tab. 1). The two dimensions NMDS of these two spectral window combinations showed that spectral windows III and V (Fig. 4a) resulted in a better separation of strains than spectral windows IV and V (Fig. 4b).

**Figure 5:**
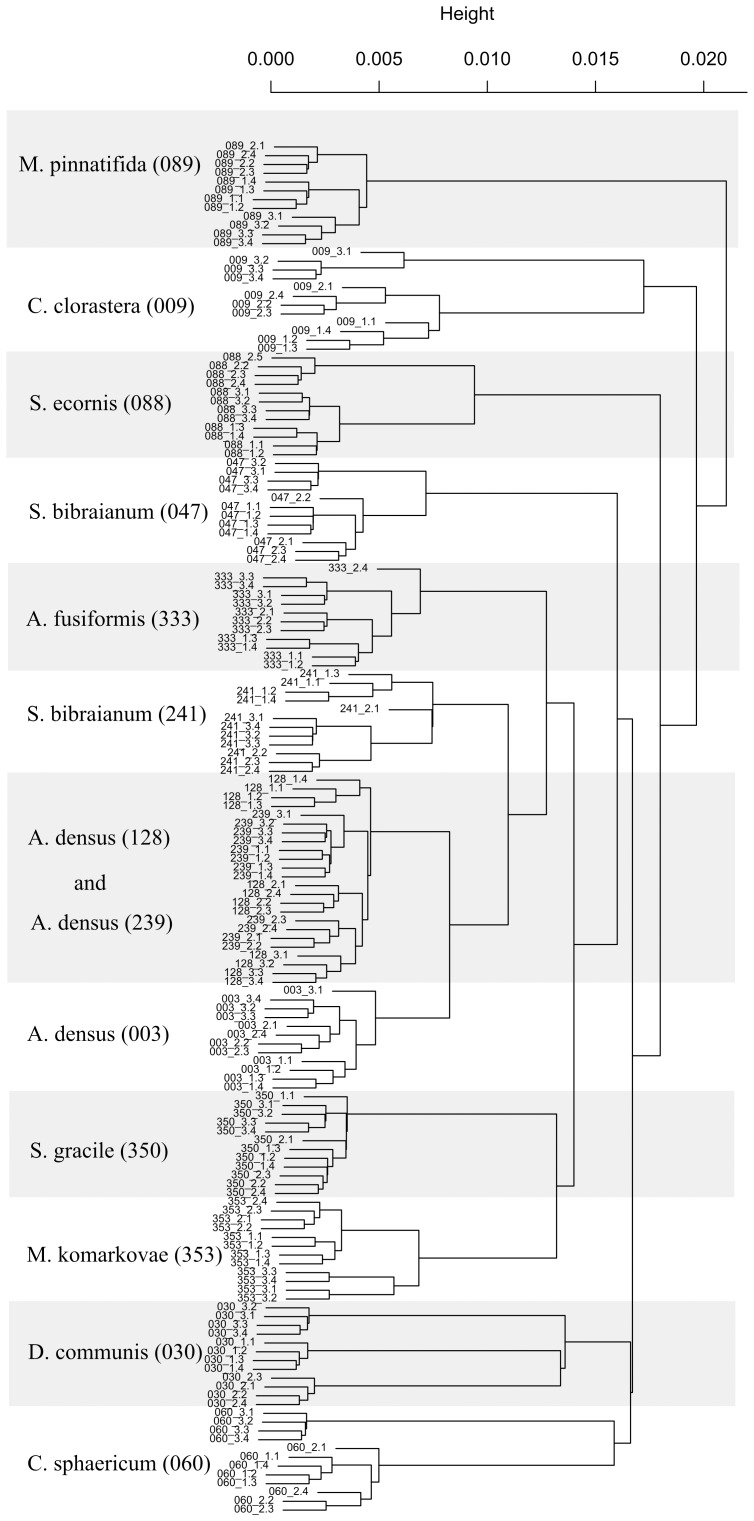
Dendrogram generated by hierarchical cluster analysis (HCA) of the strains, based on data from windows III and V of the 9-point first derivative spectra (1500–1200 cm^−1^ and 900–675 cm^−1^). 1000 replicate bootstrap values are shown, except for nodes discriminating film replicates. Strains of microalgae: *Ankistrodesmus densus* (003), *Chlamydomonas clorastera* (009), *Desmodesmus communis* (030), *Selenastrum bibraianum* (047), *Coelastrum* cf *sphaericum* (060), *Scenedesmus ecornis* (088), *Micrasterias pinnatifida* (089), *Ankistrodesmus densus* (128), *Ankistrodesmus densus* (239), *Selenastrum bibraianum* (241), *Ankistrodesmus fusiformis* (333), *Selenastrum gracile* (350) and *Monoraphidium komarkovae* (353).

**Figure 6:**
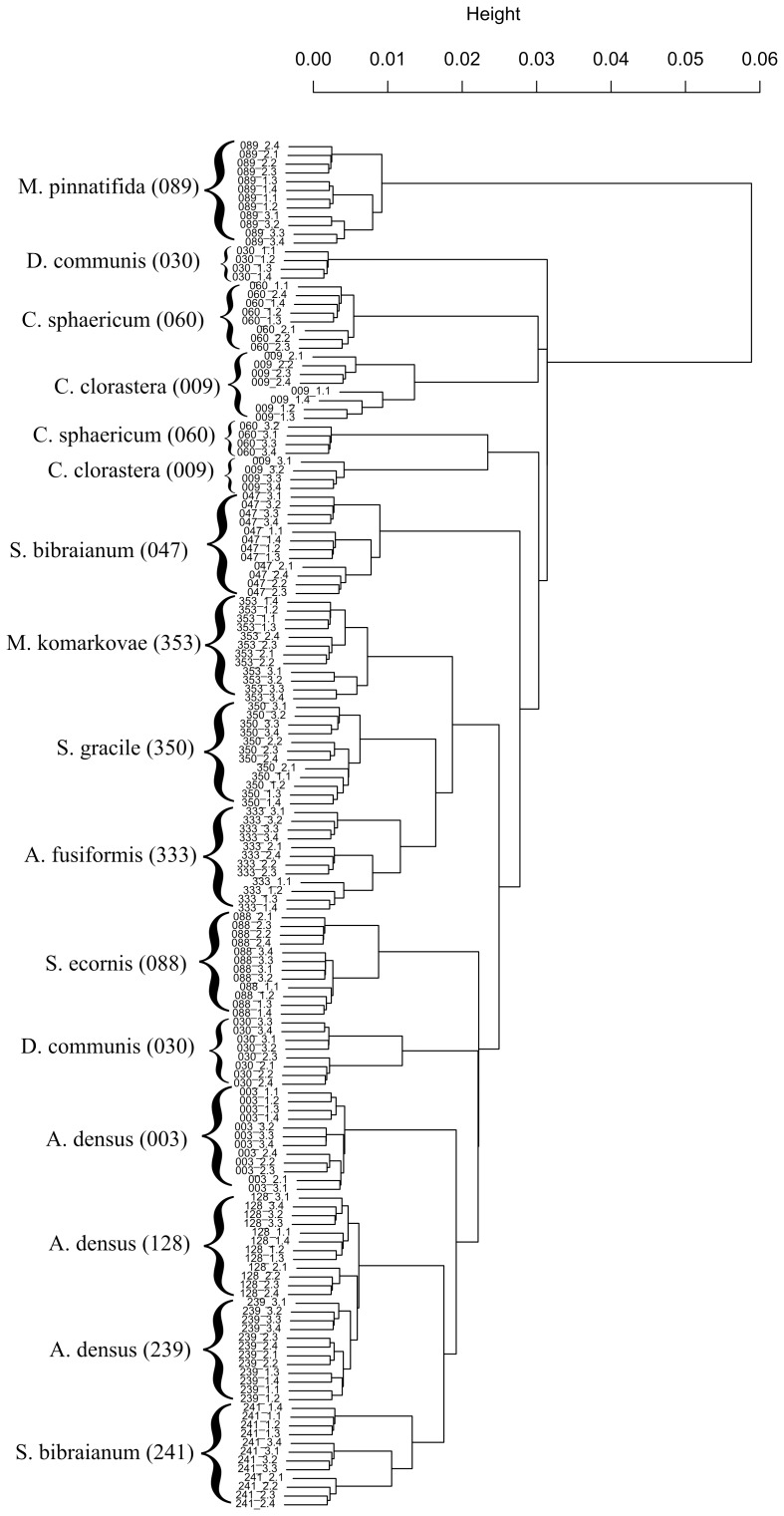
Dendrogram generated by hierarchical cluster analysis (HCA) of the strains, based on data from windows IV and V of the 9-point first derivative spectra (containing the carbohydrate window - 1200–675 cm^−1^). 1000 replicate bootstrap values are shown, except for nodes discriminating film replicates. Strains of microalgae: *Ankistrodesmus densus* (003), *Chlamydomonas clorastera* (009), *Desmodesmus communis* (Hegewald) Hegewald 2000 (030), *Selenastrum bibraianum* (047), *Coelastrum* cf *sphaericum* (060), *Scenedesmus ecornis* (088), *Micrasterias pinnatifida* (089), *Ankistrodesmus densus* (128), *Ankistrodesmus densus* (239), *Selenastrum bibraianum* (241), *Ankistrodesmus fusiformis* (333), *Selenastrum gracile* (350) and *Monoraphidium komarkovae* (353).

The combination of windows III and V also resulted in better discriminations in the HCA analysis (Fig. 5). This combination of windows provided a complete discrimination of the strains studied, with no overlap, whereas when window IV (carbohydrate region) was added, some of the replicates of strains *C. clorastera* (009), *D. communis* (030) and *C.* cf. *sphaericum* (060) were not clustered together (Fig. 6).

Concerning the hierarchical classification of the groups, the best results were achieved with windows III and V (Fig. 5), and it was in very good agreement with the current classification, although one species, *S. ecornis* (088), was positioned outside the cluster where it would be expected to be, together with strains *D. communis* (030) and *C.* cf. *sphaericum* (060).

As representatives of the order Sphaeropleales (*sensu* Krienitz & Bock [2]), we used eleven strains from six genera, divided into two families: Selenastraceae and Scenedesmaceae. In the family Selenastraceae, we have strains *A. densus* (003), *A. densus* (128), *A. densus* (239), *A. fusiformis* (333), *S. bibraianum* (047), *S. bibraianum* (241), *S. gracile* (350) and *M. komarkovae* (353). In Scenedesmaceae, we have *C.* cf *sphaericum* (060), *D. communis* (030) and *S. ecornis* (088).

All strains were discriminated almost exactly as they should, based on molecular markers (*sensu* Krienitz & Bock [2]), with one exception (*S. ecornis* 088) (Fig. 5). Considering the strains of the order Sphaeropleales, they were positioned in one greater clade that subdivides in two smaller ones plus a cluster containing only the strain *S. ecornis* (088). This greater clade could be related to order hierarchical rank, and its two subdivisions could be interpreted as the two cited families. Strain *C. clorastera* (009), presently classified in Chlamydomonadales, sister order of Sphaeropleales, was separated in a different clade, in consistency to current sistematics, and strain *M. pinnatifida* (089) was farthest positioned, as it is from a different divison (Charophyta, class Zygnemophyceae).

It is noteworthy that 3 strains of the same species, *A. densus* 003, 128 and 239, are the closest strains in the analysis, and although there is a clear distinction between strain 003 and the other two, strains 128 and 239 have some overlap. Conversely, the two strains of *S. bibraianum* (047 and 241) did not cluster together (Fig. 5).

With this carbohydrate window include in the chemometrics analysis, i.e. spectral windows IV and V, the lowest stress value in 3 dimension NMDS representation was achieved, but it also resulted in the lowest R-value of ANOSIM (Tab. 1) and a poor clustering of culture replicas were obtained with HCA analysis (Fig. 6).

## Discussion

According to the Lambert-Beer law, the absorbance of a sample is dependent on its molar absorptivity, the path length of the radiation through the sample and the concentration of the sample. Sample thickness variation introduces errors by changing the path length. Since we used the same sample concentrations in our films, and the molar absorptivity can be regarded as constant for our samples, the only source of variation, other than the variation in the cellular biochemical composition of each strain, would be the differences in the path length. However, the ATR technique removes these variations, ensuring that highly reproducible spectra are obtained.

The ATR technique eliminates this source of error because it ensures that the path length of the radiation passing through the sample is always the same. The principle of the ATR technique is that the infrared beam of the spectrophotometer enters the ZnSe crystal at a specific angle of incidence and is reflected a few times between its parallel faces. Every time one crystal face reflects the radiation it produces an evanescent wave. When the crystal face in contact with the sample reflects the radiation, the evanescent wave, an attenuated portion of the radiation beam, interacts with the sample always penetrating a fixed depth in it, and then being reflected to the other face. According to the angle of incidence and length of the crystal, a given number of internal reflections and interactions with the sample occur, and an absorption spectrum can be recorded.

The advantages of this technique is that a non-homogeneous top side of a sample has no impact on the analysis, as long as the film covers the crystal evenly, and the possibility of band saturation is greatly minimized by the micrometric penetration of the sample. Film thickness does not affect the analysis either, especially if spectra are pre-treated, for instance by differentiation, which removes differences due to varying biomass, which are represented in the raw spectra and hampers the use of the untreated data [25].

Because cell contents change during the growth of the culture, with quantities and profiles of storage compounds such as carbohydrates and lipids increasing and changing towards the later growth phases, the storage compounds are regarded as highly variable [29]–[32].

Previous works that employed FTIR technique to study microalgae showed that there is sufficient variation in the spectra to discriminate between same species being cultured with different sources of nutrients or submitted to starvation of nitrogen or phosphorus [16], [17], [19], [20]. These variations in spectra were mainly present inside the spectral region of storage compounds, showing that this high variation assessed by other techniques is captured in the spectra.

In a study with 16 marine microalgae species from different high taxa, it was found that the percentage of dry weight of these biomolecules fluctuated significantly among strains, both between and within divisions and classes [33]. For this reason, Kansiz et al [15] suggested that cultures should be sampled in the late-exponential phase, in order to minimize intra-culture variations that occur when the sampling is happens in different growth phases.

An extensive survey of lipids in microalgae in which Gas Chromatography – Mass Spectroscopy (GC-MS) analysis was used, reported that lipid profiles vary greatly, even from one study to another, and this was attributed to differences in culture conditions [34]. Overall, it was considered that lipid profiles reflect the phylogeny on higher ranks, such as divisions and classes, but that it is not an useful marker to discriminate genera and species [34]. This finding is in agreement with Harwood & Guschina [32], who said that, owing to the variable morphologies and habitats of the group, lipid contents could be extremely variable, even between algae from the same divisions.

We have tested only one of the two spectral regions attributed to lipid content: 1745–1715 cm^−1^, which is located inside window I (the other one, not tested here, being around 2920 cm^−1^). Including these spectral data impoverished our discrimination, with more strains overlapping when compared to the combination of windows III and V, in accordance to previous reports of [32], [34].

Concerning carbohydrates, in the survey of marine microalgal cited above [33], the authors reported that over 90% of the total intracellular carbohydrate were polysaccharides, and its composition did not had a pattern that could be used to discriminate taxa. With FTIR analysis, a more extensive study using complex informatics processing was conducted to determine which pre-treatment of FTIR spectral data sets and selection of variables (wavenumbers) could improve chemometrics analysis for the discrimination of two bacterial serotypes [35]. The chosen pre-treatment involved first derivatives and the selection of variables, indicating a narrow range of wavenumbers, predominantly in the region of carbohydrates, but not all wavenumbers were used, indicating that this region indeed had a high variability.

Other authors showed that changes in nutritional conditions, such as nitrogen source or Fe availability, affect the distribution pattern of species in a cluster, enabling a differentiation of not only the species, but between these conditions, in a practical use of the FTIR technique for an ecological approach [16], [26], [36]. These authors too encompassed the storage compounds spectral region in their analysis.

Our results indicate that the carbohydrates spectrum region was not effective in discriminating our strains because variations can be a physiological response to culture conditions and not a constant distinctive feature between species. In those works that differentiated both nutritional conditions of the environment and species of microalgae [16], [26], [36], the spectral storage compounds variations were a diacritic feature, necessary to achieve this discrimination; here, we removed these variations and focused on more constant features in order to correctly discriminate strains and avoid groupings derived from those physiological responses to the environment.

It has to be noted that in Preisner et al [35] only one bacterial species was utilized, with the goal of distinguishing between different serotypes. Conversely, our primary goal was to discriminate between several closely related species/strains of microalgae. Furthermore, the separation between strains was not satisfactory when window IV (related to carbohydrates) was added to our chemometrics analysis (Figs. 4b and 6). In the HCA analysis including this window (Fig. 6), not only the more aged cultures were not grouped with their respective replicas (*C. clorastera* [009_3], *D. communis* [030_1] and *C.* cf. *sphaericum* [060_3]), but the correct separation into families and orders was not achieved, *S. ecornis* (088) and *D. communis* (030) being placed in the family Selenastraceae. These findings indicate that using FTIR spectral regions related to lipids and carbohydrates for the discrimination of strains is probably not the best approach.

In our study, differences between replicates taken in the late-exponential and early-stationary growth phases were obvious in the HCA analysis (Fig. 5). Although strains were correctly distinguished from one another, the strains that had one early-stationary culture replicate analyzed - *C. clorastera* (009), *D communis* (030) and *C.* cf. *sphericum* (060) – or a replica that did not reach the late-exponential phase – *S. ecornis* (088) - clustered these replicates at a distance from the rest of the group. These four culture replicates were of different aged cultures, verified by *individual* growth curve of each culture replica (data not shown). This variation was minimized removing the spectral window regarding the storage compound carbohydrate, which tend to rise in nutrient stress situations [29] that determine the beginning of stationary phase.

Therefore, we concluded that in order to achieve a good discrimination of strains and also to obtain a reliable clustering that better reflect the classification of the group as a whole, it is better to remove the spectral regions related to storage compounds from the chemometric analysis (Fig. 5). The best discrimination and hierarchical clustering was achieved by using only the regions of true fingerprinting and the superimposed information about C = O bonds of organic acids, phosphodiesters and amide III of proteins (window V and window III, respectively, Fig. 1b).

The choice of spectral windows could indicate that adequate variable selection depends on the aim of the study: perhaps when the goal is to separate even more closely related organisms, such as different bacterial serotypes, or to probe the nutritional status of the environment, more variable regions could be more useful. The more effective spectral windows in separating the strains tested here could be tentatively assigned to cellular structural compounds that still retain sufficient variability to discriminate the organisms, as opposed to proteins spectral region (window II), which seems to be too conservative across the organisms and worsened the analysis when included.

The true fingerprint region of the spectrum (window V) arises from coupled vibrations for such as those for molecules carbon backbone [27], and it depends on associated ligands. For window III, there are absorption bands related to phosphodiesters, highly electronegative and strong covalent bonds that link consecutive pentoses in DNA and RNA strands whose high electronegativity would produce a strong absorption band in the spectrum. This electronegativity is balanced by positively charge compounds, such as histones and polyamines, which relate to the DNA packing. Therefore, analogously to the carbon backbone vibration coupling, coupling of phosphodiesters could occur, varying with the to DNA packing ratio, which would explain why this region provided good discrimination between strains. However, further research is necessary to verify this hypothesis.

Excellent discrimination between species and genera was obtained, except for the closely related strains of the same species (Fig. 5), *A. densus* strains 128 and 239. Interestingly, the strain *A. densus* (003) has been kept in our culture bank since 1979, while strains *A. densus* 128 and 239 were isolated and identified in 2009 and 2010, respectively. This observation could indicate that strains maintained in culture collections for long periods adapt to culture conditions and deviate to some degree from wild strains, while still being very close to them.

The cluster analysis generated using spectral windows III and V also reflects the currently accepted classification of these organisms with some slight differences (Fig.5), with the organisms distributed in clusters that correctly relate do order and family, except for *S. ecornis* (088), which was positioned in a cluster of its own, still inside of Sphaeropleales order but separated from Scenedesmaceae. It was also on this basis that we determined which region had the best result. Despite the fact that classification of this group has been changed frequently in the past years with the introduction of the molecular approach in addition to the morphospecies concept (see [2]), one possible reason for the positioning of *S. ecornis* (088) in our work could be that Scenedesmaceae family is somewhat sub-sampled. Our work has only three strains of Scenedesmaceae, and with more strains of this family we would expect to group all of them together in the same cluster, like what actually happened with the Selenastraceae family, which in spite of being equally scattered in NMDS (Fig. 4a) are located in one robust cluster in HCA (Fig. 5).

Within Scenedesmaceae there are some interesting observations about possible relationships between genera. Using the ITS2 marker gene, Hegewald et al [6] verified that Scenedesmaceae is subdivided in three sub-families, namely Desmodesmoidea, Coelastroidea and Scenedesmoidea, the last two sub-families being more closely related. Conversely, in our analysis we observed that strains *Desmodesmus communis* (030) and *Coelastrum* cf. *sphaericum* (060) (species from Desmodesmoidea and Coelastroidea respectively) were closer to each other than to strain *Scenedesmus ecornis* (088) (Scenedesmoidea) (Fig. 5). However, we indeed had too few strains to state this with confidence, and a more focused study on this family would be necessary to know if the relationships we found for the group through FTIR analysis were consistent.

A similar pattern of strain separation is observed in Selenastraceae. In this family, our data clustered together the three strains of *A. densus* species close to *S. bibraianum* (241) and *A. fusiformis* (333), but united these strains in a different cluster from the one containing *S. gracile* (350) and *M. komarkovae* (353). While still within of the Selenastraceae, *S. bibraianum* (047) also occupied a cluster of its own. Tracing a parallel with the Scenedesmaceae family, this species placement, with representatives of the same genus not all together, may indicate that this family too has sub-families and possibly cryptic species that display morphological convergence. More accurate observations on this issue were reported for Selenastraceae, where morphologically distinct strains produced similar 18S rRNA sequences and morphological similar strains produced distinct molecular sequences [5], and species from different genera were mixed in the classification trees obtained [4], showing the morphological convergence and phenotypic plasticity present in the group.

## Conclusions

In order to identify and discriminate closely related microalgal strains by FT-IR is necessary the development a highly reproducible spectral acquisition method. The ATR technique proved to deliver satisfactory results, with the benefit of circumventing major difficulties identified in previous studies (e.g. non-homogeneous deposition and thickness variations of sample). Additionaly, spectral region selection is a crucial step for good discrimination. Here we tested several combinations of spectral windows within the 1800–675 cm^−1^ range treated with a 9-point first derivative, and our results indicate that the use of windows related to storage compounds did not provide good discrimination between strains. Combination of regions 1500–1200 cm^−1^ and 900–675 cm^−1^ resulted in the best discrimination for chemometric analysis, with no species overlap.

Chemometric analysis appears to correctly reflect the classification of the strains tested, positioning them in accordance with the currently accepted phylogenetic classification based on markers genes, only with minor discrepancies. This is a good indicative that ATR FT-IR approach could be used in a polyphasic framework, together with marker genes and morphologic characters, providing an additional tool to help resolving the identification and classification of the highly diverse and problematic taxa of freshwater coccoid green microalgae.

## Supporting Information

S1 Fig **Comparison of raw spectra and their 9-point first derivative treatment for all strains part 1.** *Ankistrodesmus densus* (003), *Chlamydomonas clorastera* (009), *Desmodesmus communis* (Hegewald) Hegewald 2000 (030), *Selenastrum bibraianum* (047), *Coelastrum* cf *sphaericum* (060), *Scenedesmus ecornis* (088).
(TIF)

S2 Fig **Comparison of raw spectra and their 9-point first derivative treatment for all strains part 2.** *Micrasterias pinnatifida* (089), *Ankistrodesmus densus* (128), *Ankistrodesmus densus* (239), *Selenastrum bibraianum* (241), *Ankistrodesmus fusiformis* (333), *Selenastrum gracile* (350) and *Monoraphidium komarkovae* (353).
(TIF)

S1 Data **Raw data and its 9-point first derivative presented in .xlsx file for strain** ***Ankistrodesmus densus*** **(003).**
(XLSX)

S2 Data **Raw data and its 9-point first derivative presented in .xlsx file for strain** ***Ankistrodesmus densus*** **(128).**
(XLSX)

S3 Data **Raw data and its 9-point first derivative presented in .xlsx file for strain** ***Ankistrodesmus densus*** **(239).**
(XLSX)

S4 Data **Raw data and its 9-point first derivative presented in .xlsx file for strain** ***Ankistrodesmus fusiformis*** **(333).**
(XLSX)

S5 Data **Raw data and its 9-point first derivative presented in .xlsx file for strain** ***Coelastrum*** **cf.** ***sphaericum*** **(060).**
(XLSX)

S6 Data **Raw data and its 9-point first derivative presented in .xlsx file for strain** ***Chlamydomonas clorastera*** **(009).**
(XLSX)

S7 Data **Raw data and its 9-point first derivative presented in .xlsx file for strain** ***Desmodesmus communis*** **(030).**
(XLSX)

S8 Data **Raw data and its 9-point first derivative presented in .xlsx file for strain** ***Monoraphidium komarkovae*** **(353).**
(XLSX)

S9 Data **Raw data and its 9-point first derivative presented in .xlsx file for strain** ***Micrasterias pinnatifida*** **(089).**
(XLSX)

S10 Data **Raw data and its 9-point first derivative presented in .xlsx file for strain** ***Selenastrum bibraianum*** **(047).**
(XLSX)

S11 Data **Raw data and its 9-point first derivative presented in .xlsx file for strain** ***Selenastrum bibraianum*** **(241).**
(XLSX)

S12 Data **Raw data and its 9-point first derivative presented in .xlsx file for strain** ***Scenedesmus ecornis*** **(088).**
(XLSX)

S13 Data **Raw data and its 9-point first derivative presented in .xlsx file for strain** ***Selenastrum gracile*** **(350).**
(XLSX)
